# Time course and clinical relevance of thrombocytopenia in critical illness

**DOI:** 10.1186/2197-425X-3-S1-A85

**Published:** 2015-10-01

**Authors:** N Venugopal, S Abrams, Y Alhamdi, C Downey, CH Toh, ID Welters

**Affiliations:** University of Liverpool, Liverpool, United Kingdom; Institute of Infection and Global Health, University of Liverpool, Liverpool, United Kingdom; Royal Liverpool University Hospital, Liverpool, United Kingdom; Institute of Ageing and Chronic Disease, University of Liverpool, Liverpool, United Kingdom; Royal Liverpool University Hospital, Intensive Care Unit, Liverpool, United Kingdom

## Introduction

Thrombocytopenia is a common laboratory finding among critically ill patients. Studies have shown that thrombocytopenia is associated with increased morbidity and mortality, however there is limited evidence showing increasing mortality with increasing severity of thrombocytopenia. Several risk factors, such as sepsis and surgery, have been associated with thrombocytopenia. The relationship between routine coagulation parameters and platelet count has not been explored.

## Methods

This retrospective study included Intensive Care Unit (ICU) patients admitted to the Royal Liverpool University Hospital between January 2008- January 2012. Demographic details were collected at admission along with routine blood results for the first 7 days. Thrombocytopenia was categorised according to severity (severe < 50 × 10^9^/L, moderate 50-100 × 10^9^/L, mild 100-150 × 10^9^/L during the first 7 days of ICU stay). The significance of differences between groups was analysed using one-way ANOVA for normally distributed data, Kruskal-Wallis test for non-parametric data and chi-squared test for all categorical variables. Kaplan-Meier analysis was performed to show the survival within different categories of thrombocytopenia. Spearman's correlation was used to analyse the correlation between markers of coagulation activation with platelets counts on admission to ICU.

## Results

762 patients were included in the study; 162 mildly, 131 moderately and 111 severely thrombocytopenic patients were identified. The 28-day mortality was significantly higher in severely thrombocytopenic patients (30% vs 14%, p < 0.001) compared to non-thrombocytopenic patients. Platelet count dropped in all patients within the first two to five days of critical illness, followed by a compensatory increase in platelet numbers. In severely thrombocytopenic patients the platelet count continued to fall over five days and was followed by slow improvement. Severely thrombocytopenic patients had a significantly worse outcome and a longer ICU stay compared to any other category of thrombocytopenia. Persistent thrombocytopenia over seven days was associated with mortality. There is no correlation between routine coagulation values and platelets at admission.

## Conclusions

Severely thrombocytopenic patients had an increased morbidity and mortality in this large cohort study. They took longer for improvement in platelet count and are linked with poor outcomes. Persistent thrombocytopenia is also associated with mortality. Clinicians should be more vigilant with this cohort and should promptly focus on causes of thrombocytopenia in order to guide treatment.

No Grants used for this study.

